# Supplementary material for: Illness anxiety disorder and somatic symptom disorder: Similarities and differences in health-anxious individuals
Source: PLoS One. 2026 Mar 11;21(3):e0342493. doi: 10.1371/journal.pone.0342493 (PMC12978481; doi:10.1371/journal.pone.0342493)
Supplement: S1 Table — (DOCX) [file pone.0342493.s001.docx]

**Supporting Information**

**S1 Table. Demographic characteristics of the total sample.**

|  | Total sample  (N = 118) |
| --- | --- |
|  | n (%) |
| Gender |  |
| Man or male | 15 (12.7) |
| Woman or female | 99 (83.9) |
| Non-binary or different term | 4 (3.3) |
| Ethnicity |  |
| Australian | 77 (65.3) |
| Other | 41 (34.7) |
| Birthplace Australia | 88 (74.6) |
| English primary language at home | 106 (89.8) |
| Residence in Australia |  |
| Major cities/urban | 89 (75.4) |
| Regional or remote | 29 (24.6) |
| Relationship status |  |
| Single | 37 (31.4) |
| De facto/Married | 58 (49.2) |
| Divorced/Separated/Widowed | 19 (16.1) |
| Other (i.e., partnered living apart and solo polyamorous) | 4 (3.4) |
| Employment status |  |
| Unemployed | 19 (16.1) |
| Employed full-time | 33 (28) |
| Employed part-time | 43 (36.4) |
| Stay-at-home parent | 9 (7.6) |
| Carer for family member (not children) | 2 (1.7) |
| Other (i.e., student, casual work, disability pension, retirement) | 21 (17.8) |
| Level of education |  |
| High school level | 11 (9.3) |
| Certificate/diploma | 28 (23.7) |
| University undergraduate degree | 47 (39.8) |
| University postgraduate degree | 32 (27.1) |
| Past mental health treatment | 105 (89) |
| Past treatment type |  |
| Medication | 71 (65.7) |
| Therapy with psychologist | 90 (83.3) |
| Therapy with psychiatrist | 35 (32.4) |
| Support from GP | 70 (64.8) |
| Counselling from other mental health professional (i.e., nurse, social worker) | 45 (41.7) |
| Online mental health program | 32 (29.6) |
| Over-the-counter medication (e.g., vitamins) | 25 (23.1) |
| Other (i.e., exercise, diet changes, ECT) | 4 (3.7) |
| Current mental health treatment | 67 (56.8) |
| Current treatment type |  |
| Medication | 45 (60.8) |
| Therapy with psychologist | 45 (60.8) |
| Therapy with psychiatrist | 12 (16.2) |
| Support from GP | 34 (45.0) |
| Counselling from other mental health professional (i.e., nurse, social worker) | 11 (14.9) |
| Online mental health program | 6 (8.1) |
| Over-the-counter medication (e.g., vitamins) | 16 (21.6) |
| Other (i.e., exercise, diet changes, ECT) | 5 (6.8) |
