# Supplementary material for: Illness anxiety disorder and somatic symptom disorder: Similarities and differences in health-anxious individuals
Source: PLoS One. 2026 Mar 11;21(3):e0342493. doi: 10.1371/journal.pone.0342493 (PMC12978481; doi:10.1371/journal.pone.0342493)
Supplement: S2 Table — (DOCX) [file pone.0342493.s002.docx]

**Supporting Information**

**S2 Table. Nature and course of health anxiety of the total sample.**

|  | Total sample  (N = 118) |
| --- | --- |
|  | n (%) |
| Illness fears |  |
| Feared same illness | 19 (16.1) |
| Feared multiple illnesses | 99 (83.9) |
| Number of episodes |  |
| 1-7 episodes | 53 (44.8) |
| Greater than 7 episodes | 65 (55.1) |
| Total lifetime duration (health anxiety) |  |
| < 2 years | 33 (28.0) |
| 2-4 years | 18 (15.3) |
| Greater than 4 years | 67 (56.8) |
| Illness anxiety subtype |  |
| Care-seeking subtype | 34 (28.8) |
| Care-avoidant subtype | 6 (5.1) |
| Fluctuate between care-seeking and care-avoidance | 74 (62.7) |
| None of the above | 4 (3.4) |
