# Supplementary material for: Illness anxiety disorder and somatic symptom disorder: Similarities and differences in health-anxious individuals
Source: PLoS One. 2026 Mar 11;21(3):e0342493. doi: 10.1371/journal.pone.0342493 (PMC12978481; doi:10.1371/journal.pone.0342493)
Supplement: S3 Table — (DOCX) [file pone.0342493.s003.docx]

**Supporting Information**

**S3 Table. Symptom severity, quality of life, and service utilization in the total sample.**

|  | Total sample  (N = 118) |
| --- | --- |
| Symptom severity | M (SD) |
| Health anxiety (SHAI-18) | 29.9 (7.5) |
| Somatic symptoms (PHQ-15) | 14.2 (4.9) |
| Depression (PHQ-9) | 12 (6.5) |
| Generalized anxiety (GAD-7) | 10.4 (5.3) |
| Quality of life |  |
| REQoL-10 | 20.7 (7.2) |
| SF-12 Mental | 35 (9.1) |
| SF-12 Physical | 41 (12.1) |
| Service Utilization | (n = 115)* |
| Total appointments | 11.4 (14) |
| General practitioner | 3.2 (3.2) |
| Psychologist | 2.1 (3.8) |
| Psychiatrist | 0.9 (4.1) |
| Medical specialists | 1.3 (2.1) |
| Other health practitioners (i.e., physiotherapist, massage therapists, chiropractor) | 3.8 (8.7) |

*Note. Due to experimenter error, three participants did not complete the service utilization section of the survey.
