# Supplementary material for: Illness anxiety disorder and somatic symptom disorder: Similarities and differences in health-anxious individuals
Source: PLoS One. 2026 Mar 11;21(3):e0342493. doi: 10.1371/journal.pone.0342493 (PMC12978481; doi:10.1371/journal.pone.0342493)
Supplement: S4 Table — (DOCX) [file pone.0342493.s004.docx]

**Supporting Information**

**S4 Table. Health anxiety comorbidities with other mental health disorders and physical health conditions in the total sample.**

|  | Total sample  (N = 118) |
| --- | --- |
|  | n (%) |
| DSM-5 diagnoses |  |
| Generalized anxiety disorder | 53 (44.9) |
| Panic disorder | 15 (12.7) |
| Agoraphobia | 23 (19.5) |
| Obsessive compulsive disorder | 41 (34.7) |
| Major depressive disorder | 34 (28.8) |
| Somatic Symptom Disorder | 58 (49.2) |
| Illness anxiety disorder (current) | 39 (33.1) |
| Chronic illness | 56 (47.5) |
| Current chronic illness |  |
| Asthma | 15 (22.4) |
| Cancer | 3 (4.5) |
| Heart disease, stroke, or vascular disease | 5 (7.5) |
| Circulatory condition | 3 (4.5) |
| Muscular-skeletal disorders (Gout, rheumatism, osteoporosis or arthritis) | 14 (20.9) |
| Diabetes | 7 (10.4) |
| Back problems | 13 (19.4) |
| Chronic pain problems | 9 (13.4) |
| Autoimmune diseases | 10 (14.9) |
| Gynaecological disorders | 4 (6.0) |
| Other (i.e., eye conditions, connective tissue disorder, inflammatory disease) | 12 (17.6) |
