# Supplementary material for: Illness anxiety disorder and somatic symptom disorder: Similarities and differences in health-anxious individuals
Source: PLoS One. 2026 Mar 11;21(3):e0342493. doi: 10.1371/journal.pone.0342493 (PMC12978481; doi:10.1371/journal.pone.0342493)
Supplement: S5 Table — (DOCX) [file pone.0342493.s005.docx]

**Supporting Information**

**S5 Table. Demographic information of participants with current DSM-5 IAD and modified IAD diagnoses.**

|  | IAD current  (n = 39) | IAD modified  (n = 38) | IAD current vs  IAD modified |  |
| --- | --- | --- | --- | --- |
|  | n (%) | n (%) | Statistic | OR(95%CI) |
| Gender |  |  | χ^2^ (2) = 3.97, p = 0.14 |  |
| Man or male | 7 (17.9) | 3 (7.9) |  | 1.0 |
| Woman or female | 30 (76.9) | 35 (92.1) |  | 2.72 (0.65-11.46) |
| Non-binary or different term | 2 (5.2) | 0 (0.0) |  | - |
| Ethnicity |  |  | χ^2^ (2) = 3.08, p = 0.08 |  |
| Australian | 29 (74.4) | 21 (55.3) |  | 0.43 (0.16-1.12) |
| Other | 10 (25.6) | 17 (44.7) |  | 1.0 |
| Birthplace Australia | 29 (74.4) | 25 (65.8) | χ^2^ (1) = 0.68, p = 0.41 | 0.58 (0.21-1.57) |
| English primary language at home | 35 (89.7) | 31 (81.6) | χ^2^ (1) = 1.05, p = 0.31 | 0.61 (0.16-2.36) |
| Residence in Australia |  |  | χ^2^ (1) = 0.23, p = 0.63 |  |
| Major cities/urban | 29 (74.4) | 30 (78.9) |  | 1.29 (0.45-3.76) |
| Regional or remote | 10 (25.6) | 8 (21.1) |  | 1.0 |
| Relationship status |  |  | χ^2^ (3) = 5.57, p = 0.14 |  |
| Single | 11 (28.2) | 15 (39.5) |  | 2.34 (0.84-6.48) |
| De facto/Married | 24 (61.5) | 14 (36.8) |  | 1.0 |
| Divorced/Separated/Widowed | 4 (10.3) | 11 (29) |  | 3.43 (0.87-13.48) |
| Other (i.e., partnered living apart and solo-polyamorous) | 0 (0.0) | 1 (2.6) |  | - |
| Employment status |  |  |  |  |
| Unemployed | 7 (17.9) | 3 (7.9) | χ^2^ (1) = 1.72, p = 0.19 | 0.39 (0.09-1.65) |
| Employed full-time | 16 (41) | 8 (21.1) | χ^2^ (1) = 3.58, p = 0.06 | 0.38 (0.14-1.05) |
| Employed part-time | 14 (35.9) | 16 (42.1) | χ^2^ (1) = 0.31, p = 0.58 | 1.30 (0.52-3.25) |
| Stay-at-home parent | 3 (7.7) | 3 (7.9) | χ^2^ (1) = 0.00, p = 0.97 | 1.30 (0.19-5.45) |
| Carer for family member (not children) | 1 (2.6) | 0 (0.0) | χ^2^ (1) = 0.99, p = 0.32 | 0.00 (0.00-0.00) |
| Other (i.e., student, casual work, disability pension, retirement) | 2 (5.1) | 9 (23.7) | χ^2^ (1) = 5.41, p < 0.05 | 5.74 (1.15-28.65) |
| Level of education |  |  | χ^2^ (3) = 0.37, p = 0.95 |  |
| High school level | 3 (7.7) | 3 (7.9) |  | 1.0 |
| Certificate/diploma | 11 (28.2) | 9 (23.7) |  | 0.82 (0.13-5.08) |
| University undergraduate degree | 14 (35.9) | 16 (42.1) |  | 1.14 (0.20-6.60) |
| University postgraduate degree | 11 (28.2) | 10 (26.3) |  | 0.91 (0.15-5.58) |
| Past mental health treatment | 34 (87.2) | 35 (92.1) | χ^2^ (1) = 0.50, p = 0.48 | 1.72 (0.38-7.74) |
| Past treatment type |  |  |  |  |
| Medication | 22 (59.5) | 28 (80.0) | χ^2^ (1) = 3.58, p = 0.06 | 2.73 (0.95-7.85) |
| Therapy with psychologist | 30 (81.1) | 33 (94.3) | χ^2^ (1) = 2.87, p = 0.09 | 3.85 (0.74-20.00) |
| Therapy with psychiatrist | 12 (32.4) | 17 (48.6) | χ^2^ (1) = 1.95, p = 0.16 | 1.97 (0.76-5.12) |
| Support from GP | 24 (64.9) | 26 (74.3) | χ^2^ (1) = 0.75, p = 0.39 | 1.57 (0.57-4.32) |
| Counselling from other mental health professional (i.e., nurse, social worker) | 19 (51.4) | 12 (34.3) | χ^2^ (1) = 2.14, p = 0.14 | 0.49 (0.19-1.28) |
| Online mental health program | 12 (32.4) | 12 (34.3) | χ^2^ (1) = 0.02, p = 0.87 | 1.09 (0.41-2.90) |
| Over-the-counter medication (e.g., vitamins) | 8 (21.6) | 12 (34.3) | χ^2^ (1) = 1.44, p = 0.23 | 1.89 (0.66-5.40) |
| Other (i.e., exercise, diet changes, ECT) | 2 (5.4) | 1 (2.9) | χ^2^ (1) = 0.29, p = 0.59 | 0.52 (0.05-5.94) |
| Current mental health treatment | 21 (53.8) | 23 (60.5) | χ^2^ (1) = 0.35, p = 0.55 | 1.31 (0.53-3.25) |
| Current treatment type |  |  |  |  |
| Medication | 15 (53.6) | 17 (73.9) | χ^2^ (1) = 2.24, p = 0.14 | 2.46 (0.75-8.08) |
| Therapy with psychologist | 14 (50.0) | 17 (73.9) | χ^2^ (1) = 3.03, p = 0.08 | 2.83 (0.86-9.31) |
| Therapy with psychiatrist | 1 (3.6) | 6 (26.1) | χ^2^ (1) = 5.41, p < 0.05 | 9.53 (1.05-86-20) |
| Support from GP | 9 (32.1) | 13 (56.5) | χ^2^ (1) = 3.06, p = 0.08 | 2.74 (0.87-8.62) |
| Counselling from other mental health professional (i.e., nurse, social worker) | 4 (14.3) | 5 (21.7) | χ^2^ (1) = 0.48, p = 0.49 | 1.67 (0.39-7.10) |
| Online mental health program | 4 (14.3) | 1 (4.3) | χ^2^ (1) = 1.41, p = 0.24 | 0.27 (0.03-2.63) |
| Over-the-counter medication (e.g., vitamins) | 7 (25.0) | 6 (26.1) | χ^2^ (1) = 0.00, p = 0.93 | 1.06 (0.30-3.75) |
| Other (i.e., exercise, diet changes, ECT) | 2 (7.1) | 2 (8.7) | χ^2^ (1) = 0.04, p = 0.84 | 1.24 (0.16-9.55) |
