# Supplementary material for: Illness anxiety disorder and somatic symptom disorder: Similarities and differences in health-anxious individuals
Source: PLoS One. 2026 Mar 11;21(3):e0342493. doi: 10.1371/journal.pone.0342493 (PMC12978481; doi:10.1371/journal.pone.0342493)
Supplement: S6 Table — (DOCX) [file pone.0342493.s006.docx]

**Supporting Information**

**S6 Table. Nature and course of participants with current DSM-5 IAD and modified IAD diagnoses.**

|  | IAD current  (n = 39) | IAD modified  (n = 38) | IAD current vs  IAD modified |  |
| --- | --- | --- | --- | --- |
|  | n (%) | n (%) | Statistic | OR(95%CI) |
| Illness fears |  |  | χ2 (1) = 0.13, p = 0.72 |  |
| Feared same illness | 4 (10.3) | 3 (7.9) |  | 1.0 |
| Feared multiple illnesses | 35 (89.7) | 35 (92.1) |  | 1.33 (0.28-6.40) |
| Number of episodes |  |  | χ2 (1) = 0.69, p = 0.41 |  |
| 1-7 episodes | 18 (46.2) | 14 (36.8) |  | 1.0 |
| Greater than 7 episodes | 21 (53.8) | 24 (63.2) |  | 1.47 (0.59-3.66) |
| Total lifetime duration (health anxiety) |  |  | χ2 (2) = 0.46, p = 0.80 |  |
| < 2 years | 10 (25.6) | 9 (23.7) |  | 1.0 |
| 2-4 years | 7 (17.9) | 5 (13.2) |  | 0.79 (0.19-3.41) |
| Greater than 4 years | 22 (56.4) | 24 (63.2) |  | 1.21 (0.42-3.54) |
| Illness anxiety subtype |  |  | χ2 (2) = 2.43, p = 0.30 |  |
| Care-seeking subtype | 11 (28.2) | 14 (36.8) |  | 1.0 |
| Care-avoidant subtype | 2 (5.1) | 0 (0.0) |  | - |
| Fluctuate between care-seeking and care-avoidance | 26 (66.7) | 24 (63.2) |  | 0.73 (0.28-1.90) |
| None of the above | 0 (0.0) | 0 (0.0) |  | - |
