# Supplementary material for: Illness anxiety disorder and somatic symptom disorder: Similarities and differences in health-anxious individuals
Source: PLoS One. 2026 Mar 11;21(3):e0342493. doi: 10.1371/journal.pone.0342493 (PMC12978481; doi:10.1371/journal.pone.0342493)
Supplement: S7 Table — (DOCX) [file pone.0342493.s007.docx]

**Supporting Information**

**S7 Table. Symptom severity, quality of life, and service utilization of participants with current DSM-5 IAD and modified IAD diagnoses.**

|  | IAD current  (n = 39) | IAD modified  (n = 38) | IAD current vs  IAD modified |  |
| --- | --- | --- | --- | --- |
| Symptom severity | M (SD) | M (SD) | Statistic | Effect size |
| Health anxiety (SHAI-18) | 31.4 (6.4) | 32.55 (7.9) | t(75) = 0.68, p = 0.50 | d = 0.16 |
| Somatic symptoms (PHQ-15) | 13.4 (4.3) | 16.16 (5.1) | t(75) = 2.53, p < 0.05 | d = 0.58 |
| Depression (PHQ-9) | 11.3 (6.0) | 14 (6.6) | t(75) = 1.87, p = 0.07 | d = 0.43 |
| Generalized anxiety (GAD-7) | 10.8 (5.5) | 11.5 (5.5) | t(75) = 0.52, p = 0.60 | d = 0.12 |
| Quality of life |  |  |  |  |
| REQoL-10 | 20.3 (6.9) | 18.53 (6.4) | t(75) = 1.19, p = 0.24 | d = 0.27 |
| SF-12 Mental | 34.76 (9.3) | 31.41 (6.2) | t(75) = 1.86, p = 0.07 | d = 0.42 |
| SF-12 Physical | 45.7 (9.4) | 37.90 (12.8) | t(75) = 3.06, p < 0.05 | d = 0.70 |
|  |  |  |  |  |
|  | IAD current  (n = 39) | IAD modified  (n = 36)* | IAD current vs  IAD modified |  |
| Service Utilization | M (SD) | M (SD) | Statistic | Effect size |
| Total appointments | 6.87 (6.3) | 14.36 (17.2) | t(72) = 2.51, p < 0.05 | d = 0.59 |
| General practitioner | 2.3 (1.8) | 3.89 (2.5) | t(73) = 3.26, p < 0.05 | d = 0.75 |
| Psychologist | 1.39 (2.0) | 3.11 (5.1) | t(72) = 1.93, p = 0.06 | d = 0.45 |
| Psychiatrist | 0.16 (0.8) | 1.44 (6.5) | t(72) = 1.21, p = 0.23 | d = 0.28 |
| Medical specialists | 0.82 (1.6) | 1.39 (2.1) | t(73) = 1.34, p = 0.19 | d = 0.31 |
| Other health practitioners (i.e., physiotherapist, massage therapists, chiropractor) | 2.2 (4.4) | 4.53 (11.1) | t(73) = 1.23, p = 0.22 | d = 0.28 |

*Note. Due to experimenter error, two participants in the IAD modified group did not complete the service utilization section of the survey (IAD modified; n=36).
