# Supplementary material for: Illness anxiety disorder and somatic symptom disorder: Similarities and differences in health-anxious individuals
Source: PLoS One. 2026 Mar 11;21(3):e0342493. doi: 10.1371/journal.pone.0342493 (PMC12978481; doi:10.1371/journal.pone.0342493)
Supplement: S8 Table — (DOCX) [file pone.0342493.s008.docx]

**Supporting Information**

**S8 Table. Mental health and physical health comorbidities of participants with current DSM-5 IAD and modified IAD diagnoses.**

|  | IAD current  (n = 39) | IAD modified  (n = 38) | IAD current vs  IAD modified |  |
| --- | --- | --- | --- | --- |
|  | n (%) | n (%) | Statistic | OR(95%CI) |
| DSM-5 diagnoses |  |  |  |  |
| Generalized anxiety disorder | 16 (57.1) | 20 (52.6) | χ2 (1) = 0.13, p = 0.72 | 1.60 (0.65-3.93) |
| Panic disorder | 3 (7.7) | 5 (13.2) | χ2 (1) = 0.62, p = 0.43 | 1.82 (0.40-8.21) |
| Agoraphobia | 8 (20.5) | 9 (23.7) | χ2 (1) = 0.11, p = 0.74 | 1.20 (0.41-3.54) |
| Obsessive compulsive disorder | 13 (33.3) | 15 (39.5) | χ2 (1) = 0.31, p = 0.58 | 1.30 (0.51-3.31) |
| Major depressive disorder | 11 (28.2) | 14 (36.8) | χ2 (1) = 0.66, p = 0.42 | 1.49 (0.57-3.88) |
| Somatic Symptom Disorder | 11 (28.2) | 29 (76.3) | χ2 (1) = 17.85, p < 0.05 | 8.20 (2.95-22.81) |
| Illness anxiety disorder (current) | - | - | - | - |
| Chronic illness | 13 (33.3) | 21 (55.3) | χ2 (1) = 3.75, p = 0.05 | 2.47 (0.98-6.22) |
| Current chronic illness |  |  |  |  |
| Asthma | 3 (12) | 4 (20.0) | - | - |
| Cancer | 0 (0.0) | 2 (10.0) | - | - |
| Heart disease, stroke, or vascular disease | 2 (8.0) | 2 (10.0) | - | - |
| Circulatory condition | 1 (4.0) | 1 (5.0) | - | - |
| Muscular-skeletal disorders (Gout, rheumatism, osteoporosis or arthritis) | 2 (8.0) | 4 (20.0) | - | - |
| Diabetes | 2 (8.0) | 2 (10.0) | - | - |
| Back problems | 2 (8.0) | 6 (30.0) | - | - |
| Chronic pain problems | 1 (4.0) | 4 (20.0) | - | - |
| Autoimmune diseases | 3 (12) | 3 (15.0) | - | - |
| Gynaecological disorders | 0 (0.0) | 2 (10.0) | - | - |
| Other (i.e., eye conditions, connective tissue disorder, inflammatory disease) | 0 (0.0) | 8 (38.1) | - | - |

IAD current; Illness anxiety disorder, IAD Modified; Illness anxiety disorder with moderate to severe somatic symptoms, OR; Odds ratio; 95%(CI); 95% Confidence Intervals, M; mean, SD; standard deviation, 1.0; reference category, d; Cohen’s d effect size.
