# Supplementary material for: Illness anxiety disorder and somatic symptom disorder: Similarities and differences in health-anxious individuals
Source: PLoS One. 2026 Mar 11;21(3):e0342493. doi: 10.1371/journal.pone.0342493 (PMC12978481; doi:10.1371/journal.pone.0342493)
Supplement: S9 Table — (DOCX) [file pone.0342493.s009.docx]

**Supporting Information**

**S9 Table. A summary of the key research findings.**

| **Illness Anxiety Disorder compared to Somatic Symptom Disorder** | | |
| --- | --- | --- |
|  | **Non-significant** differences | **Significant** differences |
| Demographic information | No significant differences in age, gender, ethnicity, birthplace, primary language at home, residence in Australia, relationship status, most employment statuses, level of education, most past treatment types, and most current treatment types. | Individuals with SSD had:   - Lower levels of full-time employment - Higher levels of casual work - Greater past use of medication for mental health - Greater levels of current psychological therapy - More chronic health conditions, including more muscular-skeletal disorders and back problems   Compared to individuals with IAD |
| Health anxiety onset and course | No significant differences in health anxiety onset, number of health anxiety episodes, total lifetime duration, or illness anxiety subtypes. |  |
| Symptom severity | No significant differences in severity of health anxiety, generalized anxiety, depression, general quality of life, quality of life related to mental health | Individuals with SSD had more severe somatic symptoms, and lower quality of life related to physical health compared to individuals with IAD. |
| Health service use in the past three months | No significant differences in psychiatrist visits, medical specialist visits, and health practitioner visits. | Individuals with SSD had more total health care visits, more general practitioner visits and more visits to psychologists compared to individuals with IAD. |
| Comorbid mental disorders | No significant differences between IAD or SSD on all comorbid mental health disorders. |  |
| **Illness Anxiety Disorder (current) compared to Illness Anxiety Disorder (modified)** | | |
|  | **Non-significant** differences | **Significant** differences |
| Demographic information | No significant differences in age, gender, ethnicity, birthplace, primary language at home, residence in Australia, relationship status, level of education, most employment statuses, most past treatment types, and most current treatment types. | Individuals with IAD modified had:   - More likely to be in casual work - More likely to be in retirement and/or on a disability pension - More chronic health conditions - Receiving treatment from a psychiatrist at the time of the survey   Compared to individuals with IAD current. |
| Health anxiety onset and course | No significant differences in health anxiety onset, number of health anxiety episodes, total lifetime duration, or illness anxiety subtypes. |  |
| Symptom severity | No significant differences in severity of health anxiety, generalized anxiety, depression, and quality of life related to mental health. | Individuals with IAD modified had more severe somatic symptoms, and lower quality of life related to physical health compared to individuals with IAD current. |
| Health service use in the past three months | No significant differences in psychiatrist visits, medical specialist visits, and health practitioner visits. | Health service use in the past three months: total health care visits, general practitioners |
| Comorbid mental disorders | No significant differences between IAD current and IAD modified on all comorbid mental health disorders except for SSD. | IAD modified had more comorbid SSD than IAD current. |
