# Supplementary material for: Illness anxiety disorder and somatic symptom disorder: Similarities and differences in health-anxious individuals
Source: PLoS One. 2026 Mar 11;21(3):e0342493. doi: 10.1371/journal.pone.0342493 (PMC12978481; doi:10.1371/journal.pone.0342493)
Supplement: Attachment — Submitted filename: Response to Reviewers.docx [file pone.0342493.s010.docx]

**Reviewer 1**

**Comments to the author:** Thank you for the opportunity to review your manuscript. I appreciate the effort that went into this study, which addresses a critical issue in the classification and understanding of health anxiety disorders. Your work provides valuable insights into the diagnostic overlap between Illness Anxiety Disorder (IAD) and Somatic Symptom Disorder (SSD), contributing to the ongoing discussion about the validity of DSM-5 distinctions in this area. Overall, the study is methodologically sound and presents important findings; however, there are areas that require further clarification and refinement. While your results suggest minimal differences between IAD and SSD, the implications of this finding should be further explored. Additionally, there are some methodological details missing, and the presentation of results and discussion could be strengthened to better contextualize the findings in relation to previous research and clinical practice.
To enhance the clarity, rigor, and impact of the manuscript, I have outlined below several key revisions that should be addressed before the manuscript can be considered for publication.

**Response:** We appreciate your positive and helpful feedback. We have carefully considered your feedback and revised our manuscript, as outlined in our responses below.

**Abstract:**
**Comment:** Streamline verbose sentences for better readability. Ensure consistent verb tense and grammatical accuracy throughout the abstract. For ex. instead of "The objective of this paper was to compare IAD and SSD, and IAD to a modified IAD diagnosis (with moderate-to-severe somatic symptoms) in individuals experiencing health anxiety", rephrase as "This study compares IAD, SSD, and a modified IAD diagnosis (moderate-to-severe somatic symptoms) in health-anxious individuals.".

**Response:** Thank you for your suggestion. We have amended the abstract on page 2 in line with these suggestions:

Abstract, page 2:

*Little is known about the features and characteristics of illness anxiety disorder (IAD) and somatic symptom disorder (SSD), and whether the minimal somatic symptom requirement in IAD is valid and useful. This study compares IAD and SSD, and IAD (involving no or mild somatic symptoms) to a modified IAD diagnosis (modified to include moderate-to-severe somatic symptoms) in health anxious individuals. We recruited health anxious individuals drawn from the community and assessed IAD and SSD. We compared the disorders on demographic and clinical characteristics, health care utilization, and assessed the prevalence of IAD subtypes. The validity of the IAD somatic symptom criterion was examined by comparing current IAD with modified IAD. Among 118 participants, 28 met criteria for IAD, 47 for SSD, and 38 for modified IAD. Most with IAD fluctuated between seeking and avoiding medical care (71.4%), while 25% were care-seeking and 3.6% were care-avoidant. Demographic information, illness course, mental health comorbidities, and symptom severity did not differ significantly between IAD and SSD. However, individuals with SSD reported significantly more somatic symptoms, chronic health conditions, reduced quality of life, and more health care visits compared to individuals with IAD. Similar findings were observed between IAD-current and IAD-modified. The minimal differences between IAD and SSD, and between IAD-current and IAD-modified call into question the utility of distinguishing health anxiety presentations based on somatic symptom presence and severity. Further research should compare these disorders on illness course and treatment outcomes.*

**Introduction:
Comment:** Reduce redundancy in the background section: focus on the most relevant theoretical and empirical literature. Provide a more direct transition from the discussion of health anxiety to the study’s objectives. Expand on previous research that has challenged the DSM-5 distinctions between IAD and SSD and clearly explain how this study builds upon those findings.

**Response:** Thank you for your comment. We have revised the introduction (pages 3-9) to improve readability, remove redundancy, focus on the most relevant empirical research, and provide a more direct link to our study objectives and prior work challenging the DSM-5 distinctions between IAD and SSD. We are happy to make further changes if required.

**Sampling and Recruitment:**
**Comment:** Clarify how participants were screened and recruited beyond self-identification as health-anxious individuals.

**Response:** Thank you for your question. The eligibility criterion was that individuals self-identified as being anxious or worried about their health or as having health anxiety. We intentionally kept this broad with minimal criteria to incorporate a diverse community-based sample of individuals who identified as worrying about their health, expanding on existing research which typically has involved treatment seeking samples only (e.g., Newby et al., 2017). To clarify this point, we have amended page 9 (Participants and Procedure) to provide additional detail on the targeted recruitment advertisements and screening process used to determine eligibility. This includes the specific recruitment messaging, the use of an online eligibility questionnaire, and the steps participants followed to take part in the study.

Methods, page 9:

*One hundred and eighteen participants were recruited between November 2022 and 2023 through targeted online media advertisements on Instagram and Facebook. These advertisements were aimed at individuals who worried about their health or identified as having health anxiety, using messages such as “Are you suffering from health anxiety?” and “We are seeking research participants who experience persistent and frequent worries about their health…”. Interested individuals were directed to the Black Dog Institute website, which outlined the eligibility criteria and provided a link to a Qualtrics (2022) questionnaire. The questionnaire assessed eligibility, and eligible participants received an electronic participant information statement and consent form.*

**Comment:** Explain whether any efforts were made to ensure sample representativeness (e.g., gender, age, education).

**Response:** The sample was not intended to be a representative sample of the general community, as the study specifically aimed to recruit a broad sample of individuals who self-identified as experiencing health anxiety. We have transparently reported the samples’ demographic characteristics in the Results section (Table 1; page 14) and in the Supporting Information section (S1 Table; page 34), and we have acknowledged the limitations regarding generalisability in the Discussion (page 27).

Discussion, page 27:

*The present study has several limitations that need to be addressed. First, although participants were drawn from the community rather than as a treatment-seeking sample, the majority (90%) had sought treatment in the past for their general mental health concerns. Therefore, it is unclear whether our results would generalize to self-identified health-anxious individuals in the community who had not previously sought mental health treatment. We also did not ask our participants whether they sought treatment specifically for their health anxiety or for other mental health concerns in the past. In addition, our recruitment strategy required participants to self-identify with health anxiety, which may have introduced sampling bias and further limits the generalizability of our findings to the broader clinical population.*

**Diagnostic Assessment and Self-Report Measures**
**Comment:** Provide more details on interviewer training and how consistency was ensured in diagnostic assessments.

**Response:** Thank you for your suggestion. We have included more information about interview training and how consistency was maintained in the Diagnostic Interview section on pages 11 and 12. We also conducted an inter-rater reliability assessment with two blinded assessors to evaluate reliability of diagnostic groups, noted on page 12.

Diagnostic Interview, pages 11 and 12:

*The ADIS-V was administered by researchers KK and clinical psychologists EU and BC. EU and BC, already trained in administering the ADIS-V, provided training to KK. Consistency was maintained by adhering strictly to the structured interview format, using the exact wording of the script without deviation. We also assessed interrater reliability via blind ratings of audio recordings (see section below). Regular supervision with clinical psychologists and researchers AWS and JN was conducted to discuss any cases that required clarification, discussion and/or an additional rating.*

Inter-rater reliability, page 12:

*To assess inter-rater reliability, all interviews were recorded, and with participants meeting IAD or SSD criteria were assessed by an independent blinded rater. A subset of interviews were coded for the remaining diagnoses (e.g., MDD, 10%; n=18). Two clinical psychologists (BC, MH) listened to the recordings and made an independent diagnosis; these raters were blinded to the diagnosis recorded by the initial interviewer. Any discrepancies between the raters were resolved by a third rater (EU/JN). Kappa estimates between the two raters were 0.96 for IAD, 0.88 for SSD. Discrepancies for IAD and SSD arose from disagreements regarding the severity of somatic symptoms. Inter-rater agreement for other comorbid diagnoses was 100%, except for obsessive compulsive disorder (kappa=0.73).*

**Comment:** Provide more details on blinding procedures: were assessors blind to the study hypotheses or participant group?

**Response:** The interviewers/assessors were not blind to the study hypotheses as they were responsible for determining participants’ diagnostic groups. However, for the inter-rater reliability assessment, two clinical psychologists were blinded to the participants’ diagnostic groups, which has been specified in the inter-rater reliability section (pages 12-13).

Inter-rater reliability, pages 12-13:

*Two clinical psychologists (BC, MH) listened to the recordings and made an independent diagnosis; these raters were blinded to the diagnosis recorded by the initial interviewer.*

**Data Analysis:**
**Comment:** Explain if multiple comparison corrections (e.g., Bonferroni) were applied to avoid Type I errors.

**Response:** Thank you for your question. Multiple comparison corrections, such as the Bonferroni method, were not applied to our analyses as these adjustments are intended for planned comparisons and should be applied in advance rather than post hoc (see paper below). We have also added a point about this in the limitations section (page 28).

- Hooper, R. (2025). To adjust, or not to adjust, for multiple comparisons. Journal of Clinical Epidemiology, 180. <https://doi.org/10.1016/j.jclinepi.2025.111688>

Limitations, page 28:

*In addition, multiple comparison corrections were not applied to the analyses, as they were not specified prior to the analysis.*

**Comment:** Describe how missing data was handled in the analysis.

**Response**: There were only two instances of missing data which occurred in the health care utilization section of the questionnaire (six questions in total). We retained these participants in the analysis and included all data available from them. For the health care utilization outcomes, the reported *n* (SSD n=45) reflects only those participants who provided responses to each question (see page 21).

**Results**
**Comment:** Provide a clearer justification for the modified IAD category and whether it adds diagnostic value.

**Response:** Thank you for your suggestion. We have made changes to the introduction (pages 7-9) to clarify the rationale for assessing the modified IAD group. Comparing current diagnostic criteria for IAD involving an absence or only mild somatic symptoms, to modified IAD allows us to test the validity of IAD criterion B (i.e., minimal somatic symptom criterion). This investigation allowed us to determine whether the somatic symptom criterion provides meaningful qualitative differences between two IAD presentations. In the discussion (page 27) we now reflect on diagnostic value of IAD, in line with the reviewer’s suggestion.

Introduction pages 7-8:

*In the DSM-5, Criterion B for IAD specifies that health anxious individuals must have either no somatic symptoms or only mild ones, setting them apart from individuals who meet criteria for SSD who typically experience more pronounced physical symptoms. However, some researchers have questioned whether this strict IAD criteria is necessary and clinically useful [9], as clinical observations suggest that that some individuals with IAD present with moderate to severe somatic symptoms [9]. To date, no empirical studies have directly tested the validity of IAD Criterion B. Research is needed to compare individuals who meet current IAD criteria (i.e., no or minimal somatic symptoms) to those who meet all IAD criteria but report moderate-to-severe somatic symptoms. Comparing these groups is crucial for evaluating the validity of criterion B in the diagnosis of IAD. It will help to determine whether this criterion accurately captures how the disorder presents in real-world clinical settings or needs to be revised.*

Introduction, pages 8-9:

*The second aim of this study was to test the validity of IAD Criterion B, by examining whether health-anxious individuals who met current IAD criteria (i.e., no or mild somatic symptoms) differed from those who met all DSM-5 IAD criteria but reported experiencing moderate-to-severe somatic symptoms. We use the term ‘modified IAD diagnosis’ throughout this paper to describe the latter group.*

Discussion, page 27:

*This suggests that the presence or severity of somatic symptoms may be less important for clinicians when diagnosing an individual with IAD.*

**Comment:** Consider adding a summary table listing key findings to improve readability.

**Response:** Thank you for the suggestion. We have included a summary table in our supporting information file (see S9 Table).

**Comment:** Expand the discussion on healthcare utilization differences and their clinical relevance.

**Response:** We have expanded our discussion of the clinical relevance of healthcare utilization differences in the discussion section (page 26). However, we emphasise that these inferences remain speculative.

**Discussion, page 26:**

*These findings may suggest that higher somatic symptom severity in SSD may prompt individuals to seek more medical care than those with no or milder somatic symptoms, such as in IAD. Although speculative, interventions aimed at reducing or managing somatic symptoms could help reduce unnecessary healthcare visits, thereby lessening the burden on both the individual and the healthcare system.*

**Discussion**

**Comment:** Address why previous studies found greater differences between IAD and SSD and how your methodology may explain these discrepancies.

**Response:** Thank you for your suggestion. As noted in the discussion (page 26), the key methodological difference between our study and previous research is the sample (treatment-seeking participants vs. participants drawn from the community) and this difference is likely to account for the discrepancies. We also acknowledge that other factors may have contributed to the discrepancies, but it is difficult to determine this with certainty.

**Discussion, page 26:**

*The reasons underlying these discrepancies are unclear but may stem from sampling differences, as our study recruited health-anxious participants from the community, while past research recruited participants signing up to a treatment trial. It is possible that there may have been differences in severity or other factors which may have influenced the findings.*

**Comment:** Elaborate on clinical implications! If the distinction between IAD and SSD is minimal, what does this mean for diagnosis and treatment? Discuss alternative diagnostic models (e.g., a dimensional approach to health anxiety). Provide clearer treatment recommendations—Should IAD and SSD be approached similarly in therapy?

**Response:** Thank you for this great suggestion. We have added a dedicated ‘Clinical implications’ section in the discussion (pages 29-30), in which we elaborate on the clinical implications of our findings, provide treatment recommendations, and discuss alternative diagnostic models, such as the dimensional approach to health anxiety.

**Discussion, pages 29-30:**

*Clinical implications*

*The findings from this study have important implications for diagnosis, assessment and treatment of health anxiety. First, our results indicate that IAD is less common than SSD, suggesting that clinicians are more likely to encounter health-anxious individuals with moderate-to-severe somatic symptoms. Second, given the minimal differences observed between IAD and SSD, it may be appropriate for clinicians to consider a broader diagnosis for health-anxious individuals that is not constrained by somatic symptom severity. This would better reflect real-world clinical presentations, in which health anxiety can present across somatic symptom severity levels.*

*From a treatment perspective, cognitive–behavioural therapy (CBT) remains a well-supported intervention for health-anxious individuals [2], with past research showing that both IAD and SSD respond similarly to CBT [3]. Therefore, regardless of diagnostic label or somatic symptom severity, CBT appears to be an appropriate treatment option when health anxiety is present. However, for individuals with SSD who experience persistent and debilitating somatic symptoms in the absence of significant health anxiety (criterion B2), other interventions which target somatic symptom burden (i.e., drawn from research into treatments for functional somatic symptoms) may be more appropriate [4, 5].*

*Finally, the findings from this study highlight the limitations of the categorical diagnostic model (i.e., DSM-5), which presumes discrete boundaries between disorders and fails to adequately capture the continuum of health anxiety presentations [6]. However, dimensional models, such as the Research Domain Criteria (RDoC) framework, offer a potential alternative by focusing on underlying neurobiological (e.g., brain functioning, genetics, emotional responses) and behavioral (e.g., family environment, social and cultural context) processes [7]. In the context of health anxiety, this approach would better capture the heterogeneity of health anxiety presentations and provide a more nuanced understanding of symptoms, such as excessive checking and online health searches, and persistent rumination about physical sensations and their meanings.*

**Limitations**
**Comment:** Expand on potential selection bias. Self-identified health-anxious participants may not represent the full clinical population.

**Response:** We have now amended this section on page 28:

*In addition, our recruitment strategy required participants to self-identify with health anxiety, which may have introduced sampling bias and further limited the generalizability of our findings to the broader clinical population.*

**Comment:** Expand on cross-sectional design limitations. A longitudinal approach would provide deeper insights into the evolution of these disorders.

**Response:** We have added your suggestion to the limitations section (pages 27-28):

*Finally, the cross-sectional nature of our study precludes conclusions about how IAD and SSD evolve and change over time. A longitudinal study would offer valuable insights into the progression and trajectories of these disorders.*

**Comment:** Expand on reliance on self-report. Consider discussing how medical verification of symptoms could enhance future research.

**Response:** The self-report data in this study were complemented by gold-standard clinician diagnostic interviews, which serve as a form of medical verification commonly used in clinical practice. We are unclear if the reviewer comment might be referring to medically verifying somatic symptoms specifically. If that is the case, we followed the DSM approach, which relies on patients’ self-reported presence and severity of physical symptoms. This approach reflects the limitations of past DSM versions, where direct medical verification of symptoms was simply not possible to reliably obtain.

**General Points**
**Comment:** Refine language and phrasing for clarity, particularly in the abstract and discussion sections.

**Response:** We have refined the language and phrasing throughout the manuscript to improve clarity. If you have specific suggestions, we would be happy to address them.

**Comment:** Ensure APA formatting for citations, references, and tables.

**Response:** Our manuscript was formatted according to the journals guidelines which uses Vancouver style referencing.

**Comment:** Revise tables and figures to improve clarity and consistency in labelling and statistical reporting.

**Response:** Thank you for pointing this out. We have revised our tables and labelling of these tables to improve clarity and consistency.

**Reviewer #2:**

**Introduction**

**Comment:** The construct of modified IAD was based on the presence of somatic symptoms among people with IAD, which was highly assimilated the criteria of SSD. Such classification, without factor analysis from previous or current research, would be ambiguous in classifying people with the current purposed modified IAD or SSD. In addition, as the criteria of SSD itself in the DSM-5/DSM-5-TR includes the severity of somatic symptoms, regressors of somatic symptoms may inevitably correlate with SSD due to the natural definition of the disorder and induced biasness in the regression analysis.

The paragraph also did not specify the meaning of constructing a modified IAD from the current IAD vs SSD construct, neither theoretical nor clinical implications of such newly constructed classifications. It would require comparisons of the modified IAD with current existing SSD to illustrate such necessity of new classification.

**Response:** Thank you for your comment. In response to the issues raise, we have revised the introduction (pages 7-8) to clarify the rationale for comparing IAD current with IAD modified and how this differs from comparing IAD to SSD. These two comparisons address related but distinct questions. The first question, which compares IAD to SSD, examines, whether it is necessary to have two separate disorders for health-anxious individuals based on their somatic symptom severity. The second question, which compares IAD current to IAD modified, tests the validity of IAD criterion B (i.e., minimal somatic symptom criterion), and compares the two on a range of demographic and clinical characteristics. We have updated the paper accordingly to provide more detail about this.

Introduction, pages 7-8,

*In the DSM-5, Criterion B for IAD specifies that health anxious individuals must have either no somatic symptoms or only mild ones, setting them apart from individuals who meet criteria for SSD who typically experience more pronounced physical symptoms. However, some researchers have questioned whether this strict IAD criteria is necessary and clinically useful [9], as clinical observations suggest that that some individuals with IAD present with moderate to severe somatic symptoms [9]. To date, no empirical studies have directly tested the validity of IAD Criterion B. Research is needed to compare individuals who meet current IAD criteria (i.e., no or minimal somatic symptoms) to those who meet all IAD criteria but report moderate-to-severe somatic symptoms. Comparing these groups is crucial for evaluating the validity of criterion B in the diagnosis of IAD. It will help to determine whether this criterion accurately captures how the disorder presents in real-world clinical settings or needs to be revised.*

*The primary objective of this study was to replicate and extend previous research [8, 11, 12] in a sample of individuals drawn from the community who self-identified as experiencing anxiety about their health. We aimed to examine: (1) the validity and clinical utility of having two separate disorders for health anxiety based on somatic symptom presence and severity, and (2) the validity of IAD Criterion B. Specifically, the first aim was to investigate the features and characteristics of IAD and SSD. To achieve this, we recruited individuals with self-reported health anxiety, conducted diagnostic assessments for IAD and SSD and compared those who met diagnostic criteria for IAD and SSD on various demographic characteristics (e.g., age, gender, employment, education), clinical (e.g., symptom severity, functional impairment, and mental and physical comorbidity) and behavioral characteristics (e.g., healthcare utilization). The second aim of this study was to test the validity of IAD Criterion B, by examining whether health-anxious individuals who met current IAD criteria (i.e., no or mild somatic symptoms) differed from those who met all DSM-5 IAD criteria but reported experiencing moderate-to-severe somatic symptoms. We use the term ‘modified IAD diagnosis’ throughout this paper to describe the latter group.*

Further, we also acknowledge that the IAD modified and SSD groups are highly correlated and comorbid, as noted in the results section (page 23) and in S8 Table (n=29; 76.3% participants met criteria for comorbid IAD-modified and SSD). However, these constructs remain distinct. IAD modified focuses on persistent health anxiety in the presence of moderate-to-severe somatic symptoms, which may fluctuate, whereas SSD requires persistent somatic symptoms over a period longer than six months, and is accompanied by health-related worry about their somatic symptoms. Consequently, it is possible that some individuals may meet SSD criteria but not IAD modified criteria, and vice versa, as shown by the 23.7% of participants who did not meet both criteria (S8 Table).

Results, page 23:

*Furthermore, a majority of participants with modified IAD also met criteria for comorbid SSD and these individuals reported a greater number of conditions, such as POTS, bowel disease, eye conditions, and lung conditions, compared to participants meeting the current IAD criteria.*

**Comment:** Moreover, the flow of the introduction is recommended to be reorganized in a way to explain fully the current research rationale.

**Response:** Thank you for your suggestion. We have reorganised the introduction to better clarify the research rationale (pages 3-9).

**Measures, Statistical analyses**
**Comment:** Line 182-203: by the construct of the modified IAD, are there any interviewee determined with moderate/severe somatic symptoms but not with SSD?

**Response:** Yes, 23.7% of the participants meeting IAD modified criteria did not meet comorbid SSD criteria (see S8 table).

**Results**
**Comment:** The above questions were not reported nor addressed from the data comparison between IAD and SSD only, without the analyses between/among the newly constructed modified IAD. The question regarding the high similarity, if not congruence, of modified IAD with SSD, was not reported in the prevalence either.

**Response:** Thank you for your comment. We have clarified in the manuscript that our study has two primary aims: (1) to examine differences between IAD and SSD, and (2) to evaluate the validity of IAD Criterion B. While we acknowledge that there may be diagnostic overlap and comorbidity between modified IAD and SSD, analyses comparing these groups were not central to our research questions and therefore not included in our paper.

**Comment:** The differences between current IAD and SSD were, as expected, due to the severity or presence of somatic symptoms, which was inborn in the definition of the disorders in the DSM-5/DSM-5-TR. Similar problem exists in the comparison between IAD and modified IAD, which brings biasedness in the regression analysis.

**Response:** We agree that differences between current IAD and SSD largely reflect how the DSM defines them, particularly regarding somatic symptom severity. This was exactly the rationale for examining this comparison - to empirically examine whether these two diagnostic entities for health anxiety differ predominately on symptom severity relative to any other phenomenological characteristic or feature. The diagnostic decision made in DSM-5 to split health anxiety into two disorders appears to be largely arbitrary and our findings provide empirical evidence suggesting that combining these categories may reduce confusion for both researchers and clinicians.

**Comment:** Sample sizes in some of the regressor groups within the regression analyses are obviously small and may be underpowered to demonstrate if there is a significant effects from the variables.

**Response**: Thank you for pointing this out. We agree and have acknowledged this limitation on page 28.

Limitations, page 28:

*The study was also only powered to detect large differences and was underpowered to detect small-to-moderate between-group differences, meaning that we may not have detected differences of this size in our study.*

**Comment:** Exclusion of 11 participants who were in the group of "comorbid with IAD and SSD" was not explained.

**Response:** Thank you for your question. In order to address our research question about whether than qualitative differences exist between IAD and SSD, we excluded participants with comorbid IAD and SSD. However, the comorbidity between the disorders, highlights a limitation of the current diagnostic criteria as it is possible for an individual to experience health-related worries with minimal or no somatic symptoms (e.g., worrying about cancer in the absence of symptoms) and simultaneously have persistent somatic symptoms accompanied by health-related worry (e.g., chronic pain with concern that it indicates a serious condition such as multiple sclerosis).

**Discussion, Limitation, Conclusions**
**Comment:** Construct of modified IAD were not addressed in the latest sections, and the questions regarding the necessity of the new construct was not discussed. To demonstrate that the modified IAD exist, there may need a proof/research on whether the gaps, clinically or empirically, exist between IAD and SSD. For example, the consideration of Criterion F of IAD, the differences between modified IAD and SSD by factor analysis, etc. Some of the rationale of the construct demonstrated tautology in the design and may need to be refined, e.g., the effects by severity of somatic symptoms which was within criteria of distinguishing IAD and SSD, the homogeneity of modified IAD and SSD, etc.

**Response:** Thank you for your feedback. As discussed in our earlier responses, we have strengthened the introduction and rationale for comparing current IAD with modified IAD (see pages 8-9). Importantly, this study was designed to address two separate questions: (1) whether two distinct diagnoses based on somatic symptom severity are needed for health-anxious individuals, and (2) whether Criterion B of IAD is valid and clinically useful. The purpose of the modified IAD construct was not to propose a new diagnosis, but rather to serve as a comparison group to examine whether Criterion B should remain as currently defined or be broadened to include moderate-to-severe somatic symptoms, for consideration to inform the iteration of this diagnosis in the DSM. Further, in our discussion (page 28), we outline the clinical implications of this research question, noting that the presence or absence of somatic symptoms in individuals who otherwise meet full IAD criteria may not provide meaningful information to clinicians when diagnosing these patients.

Discussion, page 28:

*This suggests that the presence or severity of somatic symptoms may be less important for clinicians when diagnosing an individual with IAD.*

**Comment:** It would be interesting to see some of the participants comorbid with IAD and SSD, which should not be that case by their criteria (absence/mild vs presence/moderate or above somatic symptoms). The occurrence of such data may be worthy to research on whether the research gap exist within such classification.

**Response:** We agree with your comment. The presence of comorbidity between IAD and SSD illustrates a limitation of the current DSM-5 and was one of the reasons we conducted this research—to examine and highlight the minimal qualitative differences between the disorders. However, we did not report comorbidity data at an individual level in our manuscript, as this was not the primary focus of the current study.

**Comment:** It would be of interest to suggest for clinical implications of such classification and how different approaches may be suggested to handle the consequences from the different diagnoses.

**Response:** Thank you for your suggestion. We have included a new paragraph on clinical implications in the discussion (pages 29-30) which outlines the implications for the diagnostic groups, provides treatment recommendations, and considers alternative diagnostic models, such as the dimensional approach to health anxiety.
