# Supplementary material for: Illness anxiety disorder and somatic symptom disorder: Similarities and differences in health-anxious individuals
Source: PLoS One. 2026 Mar 11;21(3):e0342493. doi: 10.1371/journal.pone.0342493 (PMC12978481; doi:10.1371/journal.pone.0342493)
Supplement: Attachment — Submitted filename: Response_to_Reviewers_auresp_2.docx [file pone.0342493.s011.docx]

**Reviewer #2:** Thank you for the opportunity to review your manuscript. I appreciate the effort that went into the revision, however, there are some several previous comments not being addressed. In addition, there are some further comments (in revised manuscript with tracked changes) for the modified manuscript.

**Response:** Thank you for your helpful suggestions in improving our manuscript.

**Introduction**

**Comment:** The revise on Criterion B of IAD has been suggested but not on Criterion F. It is still lacking of theoretical support of building the IAD modified when highly similar disorders (e.g., SSD) cover the spectrum with moderate/severe somatic symptoms. More framework of the necessity of such construct should be elaborated.

**Response:** Thank you for your comment. In the present paper, our focus was on Criterion B; however, we acknowledge that Criterion F is also relevant as it refers to differential diagnosis. While the DSM intends to distinguish diagnostic categories through Criterion F, a recognised limitation of its categorical approach is that most diagnoses share overlapping symptom profiles. Therefore, while Criterion F is important for differential diagnosis, our study focused on Criterion B to examine health anxious individuals with varying somatic symptom severity, whereas SSD is a broader construct that does not always encompass individuals with health anxiety.

**Comment:** (Lines 96-151) Overall, the passage presents a coherent summary of three studies examining the diagnostic validity and clinical utility of IAD and SSD. However, there are a few minor inconsistencies and interpretive tensions that warrant clarification. First, while Bailer et al. (2005) is described as examining IAD and SSD, the study appears to have used post-hoc operationalizations based on DSM-IV data rather than DSM-5 diagnostic criteria; this distinction should be made explicit to avoid implying that the DSM-5 disorders were directly assessed. Second, the synthesis of findings suggests that differences between IAD and SSD are largely a matter of severity rather than qualitative distinction, yet the Swedish study is noted to have found additional quantitative differences after controlling for health anxiety severity. This point slightly complicates the overall conclusion and could be acknowledged as such.

**Response:** Thank you for your suggestion. In our introduction we specified that the study by Bialer et al used a post-hoc operationalisation of DSM-IV IAD and SSD diagnoses on both lines 76 and 91-94.

Introduction, line 76:

*“Bailer et al. [11] were the first to examine the clinical utility of distinguishing IAD and SSD on somatic symptoms using a ‘probable’ IAD and SSD diagnosis, which was operationalized from self-report questionnaires and clinical interviews.”*

Introduction, lines 91-94:

*“However, a limitation of this study was that IAD and SSD were operationalized using a post-hoc definition of the disorders, based on information gathered from self-report questionnaires and clinical interviews (for DSM-IV diagnoses) rather than being based on DSM-5 diagnostic criteria.”*

Thank you for making your point about the Swedish study. We have now streamlined this section to align the conclusion with the additional findings.

Introduction, lines 104-116:

*“More recently, a Swedish team aimed to replicate these results while accounting for trait health anxiety in a treatment-trial sample [12]. After controlling for trait health anxiety, they found no phenomenological differences in demographic and clinical characteristics related to health anxiety (e.g., age, gender, education, employment, anxiety sensitivity, intolerance to uncertainty, avoidance and safety behaviours), clinical course (e.g., age of onset) and visits to general practitioners or medical specialists. However, the authors found that SSD was associated with a greater number of psychologist visits, higher levels of disability, and more pain and tinnitus than IAD. These findings suggest that although minor differences between IAD and SSD remain after accounting for trait health anxiety, the overall distinction between the two diagnoses appear to have limited clinical value.”*

**Method**

**Comment:** (Lines 284-287) The statistical result of inter-rater reliability shall be back to the Result section.

**Response:** Thank you for your suggestion. We have moved the inter-rater reliability results to the results section, please see page 15 (lines 263-267).

**Previous Comment:** Exclusion of 11 participants who were in the group of "comorbid with IAD and SSD" was not explained.

**Current comment:** Thank you for your response for this comment. I do believe that your response should be included in the Method Section.

**Response:** Thank you for your feedback. We have included this in our methods section under statistical analyses. See page 10:

*“Participants meeting comorbid IAD and SSD criteria were excluded from analyses comparing IAD and SSD to address the research question of whether differences exist between the two disorders.*

**Limitation section**

**Comment: Previous comment regarding possibility of diagnostic overlap and comorbidity between modified IAD and SSD were not addressed in the limitation.**

**Response:** We have now included this comment in our limitation section on page 26.

*“High levels of comorbidity between SSD and IAD modified were also observed and should be considered when interpreting between-group comparisons.”*

**Previous Comment:** Construct of modified IAD were not addressed in the latest sections, and the questions regarding the necessity of the new construct was not discussed. To demonstrate that the modified IAD exist, there may need a proof/research on whether the gaps, clinically or empirically, exist between IAD and SSD. For example, the consideration of Criterion F of IAD, the differences between modified IAD and SSD by factor analysis, etc. Some of the rationale of the construct demonstrated tautology in the design and may need to be refined, e.g., the effects by severity of somatic symptoms which was within criteria of distinguishing IAD and SSD, the homogeneity of modified IAD and SSD, etc.

**Comment:** Thank you for your clarification regarding this comment. However, even the Criterion F of IAD is not the major research question in the current study, this should be considered to be discussed in the limitation section and possibly for further research. Without this discussion, the statement from Discussion, page 28, "This suggests that the presence or severity of somatic symptoms may be less important for clinicians when diagnosing an individual with IAD." is not solid.

**Response:** Thank you for your suggestion. We have now considered Criterion F in our discussion section on page 26.

Discussion, page 26:

*“More research is needed to examine the course and treatment response of IAD with or without somatic symptoms in various samples (i.e., treatment-seeking, primary and secondary care), as well as the role of differential DSM diagnostic criteria, such as Criterion F, in distinguishing IAD from related disorders including SSD.”*

**Previous Comment:** It would be interesting to see some of the participants comorbid with IAD and SSD, which should not be that case by their criteria (absence/mild vs presence/moderate or above somatic symptoms). The occurrence of such data may be worthy to research on whether the research gap exist within such classification.

**Comment:** Please include your response in the discussion/limitation section.

**Response:** Thank you for your suggestion. We have discussed this limitation in the limitation section on page 26.

*“Further, there were high levels of comorbidity between IAD and SSD, illustrating limitations of the current DSM-5. However, comorbidity between IAD and SSD was not reported at an individual level, as this was not the primary focus of the current study.”*
